# Urine Annexin A1 as an Index for Glomerular Injury in Patients

**DOI:** 10.1155/2014/854163

**Published:** 2014-01-20

**Authors:** Shuk-Man Ka, Pei-Yi Tsai, Tai-Kuang Chao, Shun-Min Yang, Yi-Jen Hung, Jin-Shuen Chen, Hao-Ai Shui, Ann Chen

**Affiliations:** ^1^Graduate Institute of Aerospace and Undersea Medicine, National Defense Medical Center, Taipei, Taiwan; ^2^Department of Animal Pharmacology, Development Center for Biotechnology, Taipei, Taiwan; ^3^Department of Pathology, Tri-Service General Hospital, National Defense Medical Center, Taipei, Taiwan; ^4^Division of Endocrinology & Metabolism, Department of Internal Medicine, Tri-Service General Hospital, National Defense Medical Center, Taipei, Taiwan; ^5^Division of Nephrology, Department of Internal Medicine, Tri-Service General Hospital, National Defense Medical Center, Taipei, Taiwan; ^6^Graduate Institute of Medical Sciences, National Defense Medical Center, Taipei, Taiwan

## Abstract

*Background.* We recently demonstrated high urine levels of annexin A1 (ANXA1) protein in a mouse Adriamycin-induced glomerulopathy (ADG) model. *Objective.* To establish ANXA1 as a potential biomarker for glomerular injury in patients. *Methods.* A time-course study in the mouse ADG model, followed by renal tissues and urine samples from patients with various types of glomerular disorders for ANXA1. *Results.* Urinary ANXA1 protein was (1) detectable in both the ADG model and in patients except those with minimal change disease (MCD); (2) positively correlated with renal lesions in patients; and (3) early detectable in diabetes patients with normoalbuminuria. *Conclusions.* ANXA1 is a universal biomarker that is helpful in the early diagnosis, prognostic prediction, and outcome monitoring of glomerular injury. Measurement of urinary ANXA1 protein levels can help in differentiating MCD from other types of glomerular disorders.

## 1. Introduction

Despite their well-known limitations, currently, the most widely used biomarkers for the early detection of chronic kidney disease or acute kidney injury are proteinuria, serum creatinine (Cr), and blood urea nitrogen (BUN). However, all of these indexes are less than optimal and are probably more relevant to later stages of injury, when therapeutic responses might be poor. The value of using serum Cr or albuminuria as a reliable urinary marker is being increasingly challenged in terms of its prediction value in certain renal conditions [1, 2]. In addition, regardless of the type of glomerular disorder, neither albumin nor Cr is produced in the kidney. Recently, there has been great interest in strategies aimed at identifying novel biomarkers that can be easily detected in a noninvasive way (such as the use of urine samples) and can predict renal damage during the earlier stages [3]. Glomerular disorders represent a major health care problem because of their high mortality and morbidity rates [4]. Several studies have demonstrated that glomerular disorders are a multifactorial process caused by immune and nonimmune mechanisms that lead to glomerulosclerosis, tubulointerstitial fibrosis, inflammatory infiltration, loss of renal parenchyma, and renal vascular changes [5, 6]. Although glomerular disorders can cause significant morbidity and mortality, they are treatable and constitute a preventable cause of renal failure and cardiovascular risk [7]. Importantly, the early recognition of the disease and the timely institution of appropriate treatment for patients with glomerular disorders should be beneficial.

We recently demonstrated overproduction of annexin A1 (ANXA1) in the urine in a mouse Adriamycin-induced glomerulopathy (ADG) model, featuring both chronic glomerulosclerosis and tubulointerstitial damage, by using the two-dimensional electrophoresis gel and matrix-assisted laser desorption ionization/time of flight mass spectrometry analysis [8]. We also identified another member of the annexin family, annexin A2 (ANXA2), as an early sensor of tubular injury that persists throughout the recovery process of tubular cells in acute renal failure [9] and is upregulated in both the glomerulus and renal tubules in crescentic glomerulonephritis [10]. Annexins are a family of calcium-dependent phospholipid-binding proteins that bind reversibly to membranes through calcium-binding loops in their highly conserved core domains [11, 12]. ANXA1 acts as an important endogenous anti-inflammatory molecule [13–15] and has a variety of other biological activities, including regulation of cell proliferation and cell death signaling and promoting efficient phagocytosis of apoptotic cells [13]. ANXA2 is also involved in a broad range of biological processes, such as cell migration [16], plasminogen activation [17], signal transduction [18], and resolution of inflammation [19]. Furthermore, ANXA1 or ANXA2 mRNA or protein levels are altered in various human diseases, for example, cystic fibrosis [20, 21], hepatocellular carcinoma [22, 23], prostate carcinoma [24–26], pancreatic cancer [27, 28], breast cancer [29–31], and renal cell carcinoma [32, 33]. However, the expression patterns and potential roles of ANXA1 and ANXA2 remain unclear in most glomerular disorders.

In the present study, we characterized the expression pattern and tissue distribution of ANXA1 and ANXA2 in the ADG model over time and confirmed these results in a large number of different types of human glomerular disorders. We infer that ANXA1 and ANXA2 may play important pathogenic roles in glomerular disorders. Importantly, only ANXA1, which is significantly increased in the kidney and urine, can be used as an early noninvasive urinary biomarker for most types of glomerular disorders.

## 2. Methods

### 2.1. ADG Animal Model and Experimental Protocol

ADG was induced in C57BL/6 mice by a single injection of Adriamycin as described previously [34]. Urine was collected using metabolic cages, and proteinuria and BUN and Cr levels in the serum were evaluated as described previously [35]. Mice were sacrificed at days 0, 3, 7, 14, and 21 for pathological evaluation, extraction of mRNA for real-time reverse transcription-polymerase chain reaction (RT-PCR) analysis, and detection of urinary levels of specific proteins. All animal experiments were performed with the ethical approval of the Institutional Animal Care and Use Committee of the National Defense Medical Center, Taiwan, and according to the ethical rules in the NIH Guide for the Care and Use of Laboratory Animals.

### 2.2. Renal Tissues and Urine Samples from Patients with Glomerular Disorders

Eighty-one patients with glomerular disorders, consisting of minimal change disease (MCD) (*n* = 19), IgA nephropathy (IgAN) (*n* = 10), membranous glomerulonephritis (MGN) (*n* = 12), focal segmental glomerulosclerosis (FSGS) (*n* = 11), crescentic glomerulonephritis (CrGN) (*n* = 5), diabetic nephropathy (DN) (*n* = 8), or lupus nephritis (LN) (*n* = 16), were retrospectively included in this study. Renal biopsy specimens and urine samples were obtained at the time of diagnosis. Renal tissues taken from the unaffected pole of kidneys removed because of renal cell carcinoma were used as normal controls. Normal urine samples were collected from apparently healthy volunteers (*n* = 15). The urinary albumin excretion is examined by the ratio of urinary albumin concentration to urinary Cr concentration (UACR) [36].

On the other hand, 47 urine samples from diabetic patients without albuminuria (normoalbuminuria) or with early kidney involvement (microalbuminuria or macroalbuminuria) were also included in this study. In those patients, normoalbuminuria was defined as UACR <30 mg/g, microalbuminuria was defined as UACR cutoff of 30–299 mg/g, and macroalbuminuria was defined as UACR ≥300 according to the criteria of the American Diabetes Association [37]. Detailed patient characteristics are given in Supplementary Table 1 in Supplementary Material available online at http://dx.doi.org/10.1155/2014/854163.

The patients were followed at the Tri-Service General Hospital, Taipei, Taiwan, and all patients who contributed samples signed an informed consent form, as required by the regulations of the Institutional Review Board of the Tri-Service General Hospital, National Defense Medical Center, Taipei, Taiwan.

### 2.3. Renal Pathological Evaluation

Renal tissues were fixed in 10% buffered formalin, embedded in paraffin, and stained with hematoxylin and eosin for routine histopathologic evaluation. For mice histopathologic evaluations of sclerosis, at least 50 glomeruli in renal tissue sections for each case were examined. Severity of sclerosis was semiquantified by morphological changes on a scale of 1–4, as described previously [38]. For patients, pathological parameters such as activity indices and chronicity indices were approached by renal pathologists as described previously [39] involving semiquantitative scoring of specific biopsy features. The proportion was calculated for the following four major components: (1) glomerular proliferation, (2) glomerular sclerosis, (3) interstitial inflammation, (4) interstitial fibrosis, and (5) crescentic formation, respectively. Differences in scoring between the pathologists were resolved by rereviewing the biopsies and thus reaching a consensus.

### 2.4. Real-Time RT-PCR Examination

Renal cortex RNA was extracted using TRIzol reagent (Invitrogen, CA, USA) according to the manufacturer's instructions and real-time RT-PCR was used to verify altered gene expression in the ADG animal model as described previously [34]. The primers used are shown as follows: mouse ANXA1: 5′-GGGACTTGGAACAGATGAAGAC-3′, 5′-GTCTGTCCCCTTTCTTCCTTTCT-3′; mouse ANXA2: 5′-GTGGATGAGGTCACCATTGTC-3′, 5′-GTCGGTTCCTTTCCTCTTCAC-3′; and mouse GAPDH: 5′-TCCGCCCCTTCTGCCGATG-3′, 5′-CACGGAAGGCCATGCCAGTGA-3′. Amplifications were normalized to GAPDH using the 2^−ΔΔCT^ method [40].

### 2.5. *In Situ* Hybridization (ISH)

ISH was performed as described previously [10]. Formalin-fixed and paraffin-embedded renal sections were used. The cDNAs used as templates for the synthesis of the specific RNA probes were generated from mouse kidney or human renal biopsies by RT-PCR. The primers used for mice as described in the real-time RT-PCR section and the primers used for human were ANXA1: 5′-TGAGGAGGTTGTTTTAGCTCTG-3′, 5′-GTTCTTGATGCCAAAATCTCAA-3′; ANXA2: 5′-TCTACTGTTCACGAAATCCTGT-3′, 5′-TTCAATAGGCCAAAATCACCG-3′. Scoring of mRNA expression levels in kidney tissues was performed under light microscopy and 50 randomly selected cortical fields were examined in each section as described previously [10].

### 2.6. Immunohistochemistry (IHC)

Formalin-fixed and paraffin-embedded renal sections were prepared as described previously [41] and were stained using primary antibodies against mouse ANXA1 (Zymed Laboratories, CA, USA), human ANXA1 (BD Biosciences, CA, USA), or human/mouse ANXA2 (Santa Cruz Biotechnology, CA, USA), biotinylated secondary antibody, streptavidin-conjugated horseradish peroxidase, and AEC reagent (DakoCytomation, CA, USA). Semiquantitative evaluation was performed as described above for ISH.

### 2.7. Western Blot Analysis of Urine Samples

Protein levels of ANXA1 and ANXA2 in urine samples from mice with ADG or patients were measured by Western blot analysis as described previously [10] using the antibodies described in the IHC section. The data are presented as the ratio of the density of the target protein band to the Cr concentration in the urine as described previously [10].

### 2.8. Data Analysis

The results are presented as the mean ± SEM or median. Comparisons between two groups were performed using Student's *t*-test. The correlation coefficient was Pearson's *r* values. A *P* value < 0.05 was considered statistically significant.

## 3. Results

### 3.1. Clinical Manifestations and Pathological Features of the ADG Model

Persistent heavy proteinuria and deteriorated renal function were observed in the ADG mice after a single Adriamycin injection. Compared to normal controls, the mice showed an increase in Cr-corrected urine protein levels (mg/mg) on day 7 (1.14 ± 0.11 versus 0.28 ± 0.09, *P* < 0.05), and levels remained high till the end of the experiment on day 21 (2.21 ± 0.27, *P* < 0.005). BUN levels in the serum showed a significant and progressive increase from day 14 (63.2 ± 8.12 mg/dL, *P* < 0.01) to day 21 (96.81 ± 5.42 mg/dL, *P* < 0.005) compared to normal controls (23.9 ± 2.34 mg/dL), and serum Cr levels also showed a significant increase from day 7 (0.96 ± 0.09 mg/dL versus 0.46 ± 0.04 mg/dL, *P* < 0.05) and progressively increased until the mice were sacrificed on day 21 (2.45 ± 0.18 mg/dL, *P* < 0.005).

**Figure 1:**
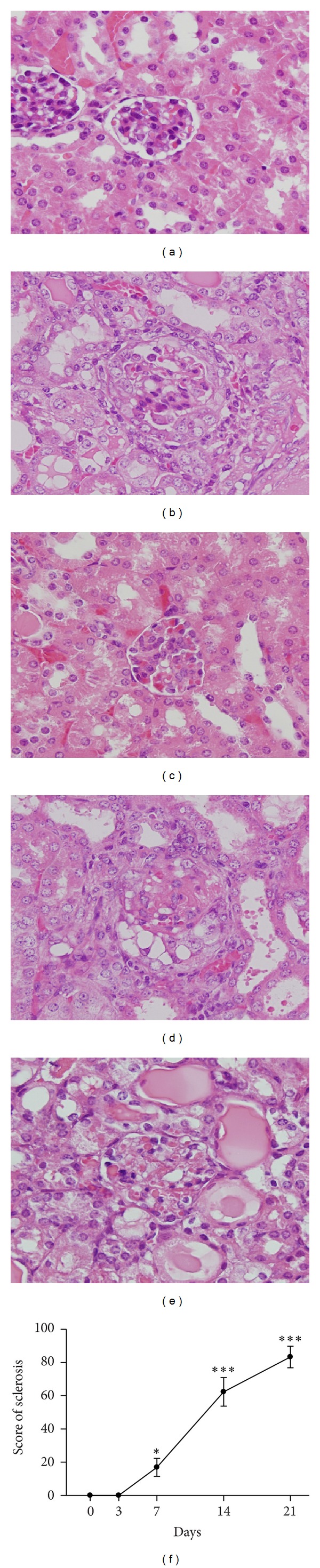
Glomerular histopathology in ADG mice. (a)–(e) Kidney tissue histopathological sections on day 0 (a), day 3 (b), day 7 (c), day 14 (d), or day 21 (e), showing a gradual increase in sclerosis in the glomeruli (hematoxylin and eosin staining). Original magnification, ×400. (f) Semiquantitative plot showing the sclerosis scores for the glomeruli in the tissue sections. Each point represents the mean ± SEM for six mice per group. *P < 0.05, ***P < 0.005 compared to the normal controls (day 0).

Histopathological examination was performed on kidney sections at different time points (Figure 1). Expansion of the extracellular matrix and deposition of hyaline mass in the glomeruli were occasionally seen on day 7 and became obvious and statistically significant from day 14 to day 21. Importantly, the mice exhibited a significant and steady increase in the percentage of glomeruli containing epithelial hyperplastic lesions from day 14 to day 21, suggesting a progressive pathological status. The sclerosis score is shown in Figure 1(f) and correlated well with the severity of the renal biological parameters.

### 3.2. Increased ANXA1 and ANXA2 mRNA Levels in the ADG Model

**Figure 2:**
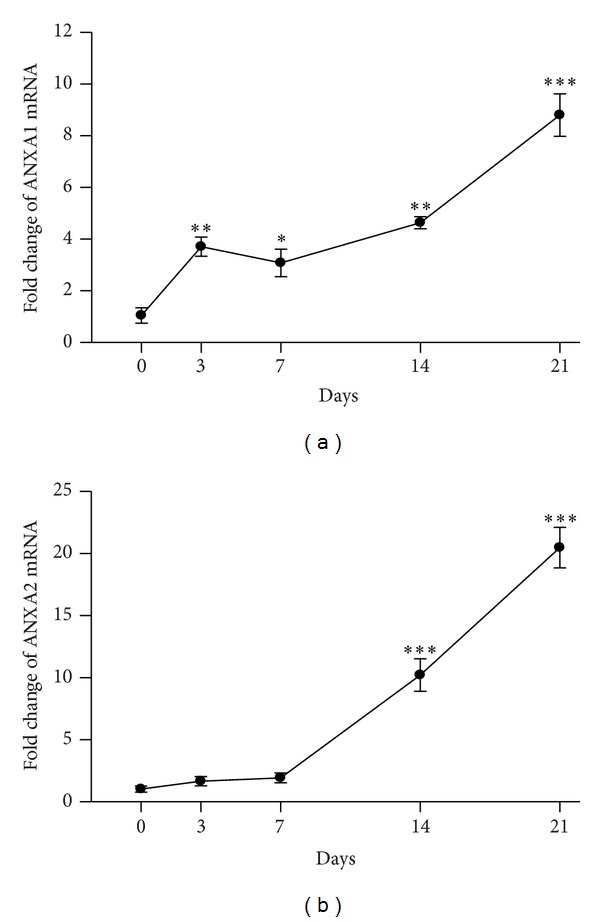
Quantitative analysis of mRNA levels in renal cortex from ADG mice by real-time RT-PCR over time. (a) ANXA1 and (b) ANXA2. Each point represents the mean ± SEM for six mice per group. *P < 0.05, **P < 0.01, ***P < 0.005 compared to the controls (day 0).

To determine whether ANXA1 and ANXA2 mRNA levels changed with disease evolution, real-time RT-PCR analysis was performed on total RNA extracted from the renal cortex at various points after ADG induction. As shown in Figure 2(a), renal ANXA1 mRNA levels were significantly increased in the ADG model as early as day 3 and remained persistently increased up to day 21 when the mice were sacrificed (day 3, *P* < 0.01; day 7, *P* < 0.05; day 14, *P* < 0.01; day 21, *P* < 0.005) compared to day 0. In contrast, as shown in Figure 2(b), ANXA2 mRNA levels only showed a significant increase on day 14 and day 21 compared to day 0 (*P* < 0.005 for both).

**Figure 3:**
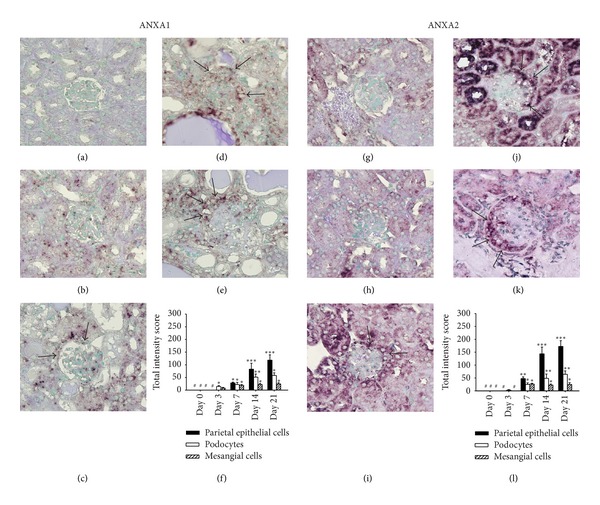
Cellular expression of ANXA1 and ANXA2 mRNAs demonstrated by ISH at different time points after ADG induction. (a)–(f) ANXA1 and (g)–(l) ANXA2. Kidney tissue sections on day 0 ((a) and (g)), day 3 ((b) and (h)), day 7 ((c) and (i)), day 14 ((d) and (j)), or day 21 ((e) and (k)). The arrows indicate epithelial cells in the crescent-like formation of the glomerulus. Original magnification, ×400. (f) and (l) Semiquantitative analysis of cellular protein expression by ISH. Scoring was performed for the three major components of the parietal epithelial cells (solid bars), podocytes (open bars), and mesangial cells (hatched bars). Each bar represents the mean ± SEM for six mice per group. *P < 0.05; **P < 0.01; ***P < 0.005, compared to the normal controls (day 0). #: not detectable.

Next, we used ISH to determine which cells in the renal tissues showed ANXA1 and ANXA2 gene expression over time. As shown in Figure 3, ANXA1 mRNA was expressed as early as day 3 (Figures 3(b) and 3(f)), predominantly in the podocytes and some renal tubules, consistent with the real-time RT-PCR findings. ANXA1 staining of the parietal epithelial cells, podocytes, and epithelial hyperplastic lesions of the glomerulus was seen from day 7 until day 21, when the mice were sacrificed, although few renal tubules were positive (Figures 3(c)–3(f)). However, only basal levels of ANXA2 mRNA were seen in the renal tubules from day 0 to day 3 (Figures 3(g), 3(h), 3(l)), with a gradual increase on day 7 (Figures 3(i) and 3(l)). On days 14 and 21 (Figures 3(j)–3(l)), the pattern of ANXA2 mRNA expression was similar to that of ANXA1 mRNA, except that ANXA2 mRNA levels were even higher than those for ANXA1 in certain renal tubules with potential regeneration activity.

### 3.3. Increased ANXA1 and ANXA2 Protein Expression in the ADG Model

**Figure 4:**
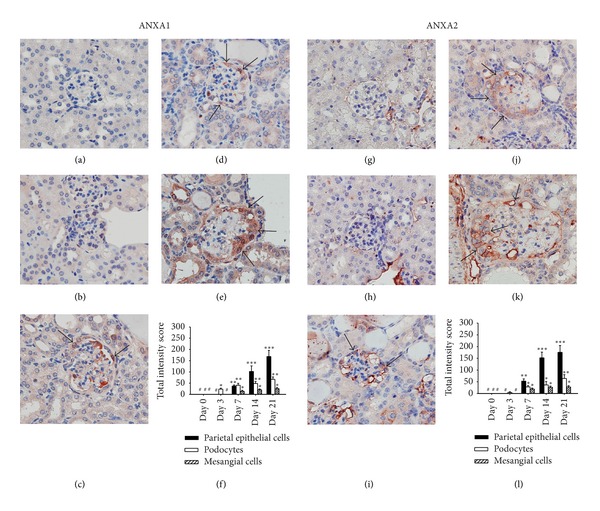
Detection of ANXA1 and ANXA2 protein expression in kidney tissue by IHC at different time points after ADG induction. (a)–(f) ANXA1 and (g)–(l) ANXA2. Kidney tissue sections on day 0 ((a) and (g)), day 3 ((b) and (h)), day 7 ((c) and (i)), day 14 ((d) and (j)), or day 21 ((e) and (k)). The arrows indicate epithelial cells in the crescent-like formation of the glomerulus. Original magnification, ×400. (f) and (l) Semiquantitative analysis of cellular protein expression by IHC. Scoring was performed for the three major components of the parietal epithelial cells (solid bars), podocytes (open bars), and mesangial cells (hatched bars). Each bar represents the mean ± SEM for six mice per group. *P < 0.05; **P < 0.01; ***P < 0.005, compared to the normal controls (day 0). #: not detectable.

The tissue and cell location of ANXA1 and ANXA2 protein expression in the kidney were evaluated by IHC. Both ANXA1 (Figures 4(a)–4(f)) and ANXA2 (Figures 4(g)–4(l)) were significantly increased as early as day 7 and till day 21, compared to day 0. Moreover, approximately 25% of the ANXA1 stained cells showed a nuclear staining pattern in addition to the cytoplasmic staining pattern.

### 3.4. Dynamic Changes in Urine ANXA1 Levels in the ADG Model

**Figure 5:**
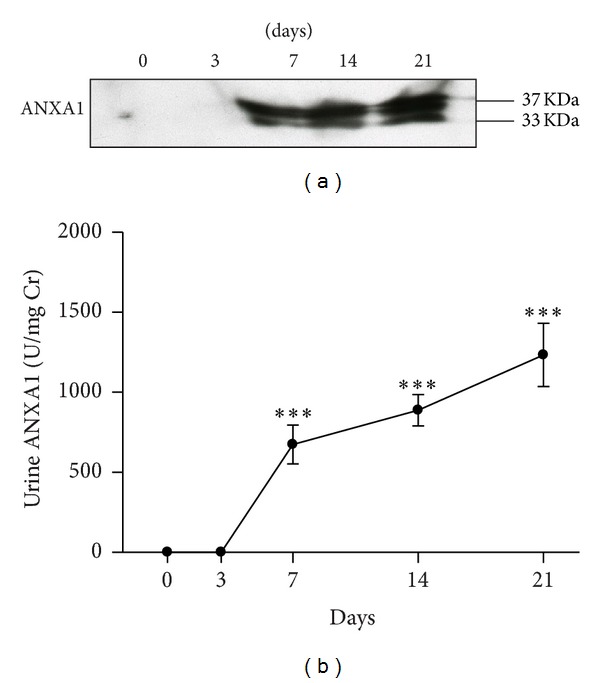
Detection of urine ANXA1 levels by Western blotting during the course of the ADG model. (a) Representative Western blot of urine samples; the molecular weights are shown on the right. (b) Quantitative analysis of the ratio of the protein band density to urinary creatinine over time. Each point represents the mean ± SEM for six mice per group. ***P < 0.005 compared to the normal controls (day 0).

We examined whether these proteins were excreted in the urine using Western blot analysis. As shown in Figures 5(a) and 5(b), ANXA1 protein was not detectable in the urine on days 0 and 3, but a significant increase was seen from day 7 to day 21 (day 7, 672.73 ± 121.06 U/mg Cr; day 14, 887.27 ± 97.65 U/mg Cr; day 21, 1232.44 ± 197.88 U/mg Cr versus day 0, 0 U/mg) (all *P* < 0.005). Importantly, both 37 kDa intact ANXA1 and 33 kDa cleaved ANXA1 were observed in the urine (Figure 5(a)). In contrast, ANXA2 was not detected in the urine in normal control mice or at any time in the ADG model.

### 3.5. Enhanced ANXA1 and ANXA2 mRNA and Protein Expression in Renal Tissues of Patients with Various Glomerular Disorders except MCD

**Figure 6:**
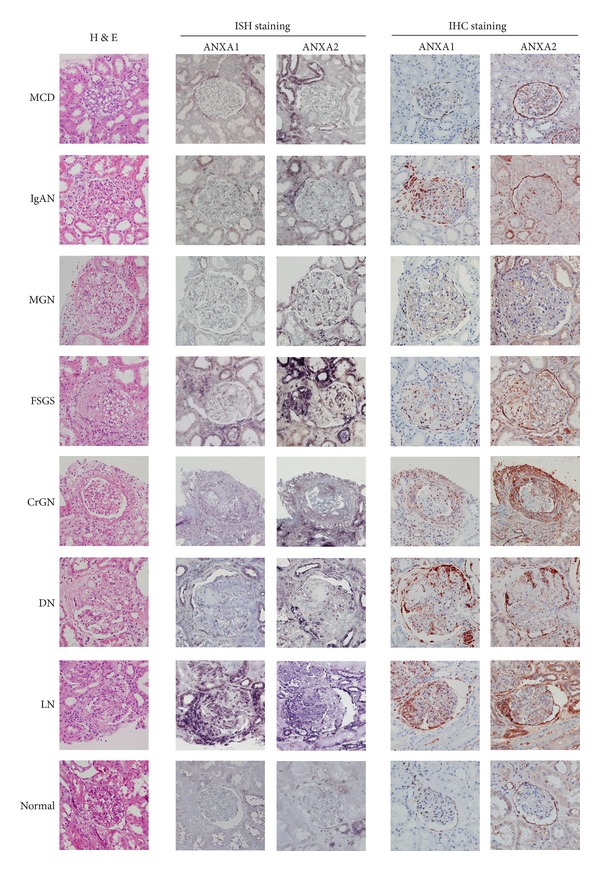
Detection of ANXA1 and ANXA2 mRNA and protein expression in kidney tissues from patients with various types of glomerular disorders by ISH or IHC. Representative histopathology of kidney tissues by haematoxylin and eosin staining. Original magnification, ×400. MCD: minimal change disease; IgAN: IgA nephropathy; MGN: membranous glomerulonephritis; FSGS: focal segmental glomerulosclerosis; CrGN: crescentic glomerulonephritis; DN: diabetic nephropathy; LN: lupus nephritis.

We then examined ANXA1 and ANXA2 mRNA and protein expression in patients with various types of glomerular disorders by ISH or IHC staining. As shown in Figure 6, although ISH showed that very little ANXA1 mRNA was expressed in the tubules or glomeruli in the normal controls (bottom line, left panels) and patients with MCD (top line, left panels), most biopsy tissues from other types of glomerular disorders were found to have high levels of ANXA1 mRNA in the glomeruli examined. The major sites of ANXA1 mRNA expression were in the podocytes and parietal epithelial cells in IgAN, MGN, and FSGS. Dense intracellular staining was seen in the fibrotic region of glomeruli, especially in the crescentic formation in CrGN, DN, and LN. In the tubulointerstitial compartment, ANXA1 mRNA expression was mainly focally localized in regenerating tubules and infiltrating inflammatory cells. ANXA2 mRNA showed a similar distribution and intensity to that of ANXA1 mRNA.

The pattern of ANXA1 and ANXA2 protein expression, as demonstrated by IHC, was generally similar to that of the mRNAs. Moreover, consistent with the results in the ADG model, approximately 25% of ANXA1-stained cells showed nuclear staining in addition to cytoplasmic staining, and ANXA2 protein was also expressed in the plasma membrane in epithelial cells in the crescentic formation.

### 3.6. Increased Urinary ANXA1 Protein Levels in Patients

#### 3.6.1. In Various Glomerular Disorders except MCD

To determine whether ANXA1 could serve as a biomarker for noninvasive diagnosis and prognostic prediction, we measured urine protein levels in patients with various types of glomerular disorders and apparently healthy donors by Western blot analysis. As shown in Table 1, ANXA1 protein was detectable in the urine of almost all patients, compared to normal controls, except those with MCD (less than 10.52% of cases were detected). Importantly, excluding MCD, urinary levels of ANXA1 were significantly positively correlated with the levels of UACR (*r* = 0.43, *P* < 0.001).

#### 3.6.2. Positive Correlation with Renal Lesions

**Figure 7:**
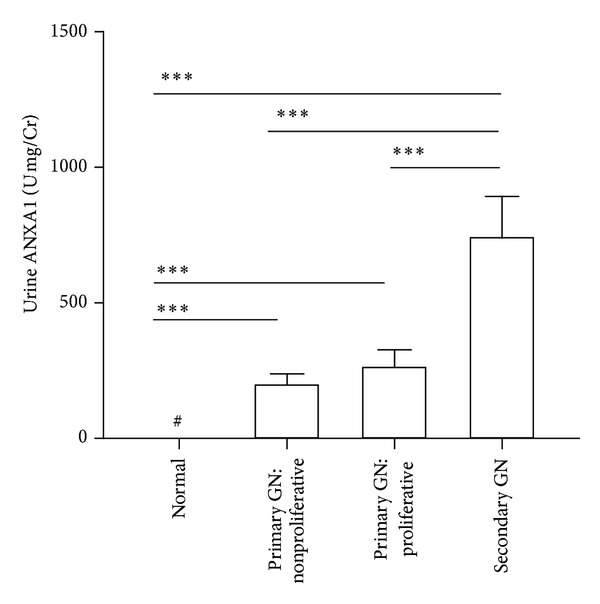
Urine ANXA1 levels correlated in patients with various types of glomerular disorders. GN: glomerulonephritis. ***P < 0.005. #: not detectable.

We then attempted to identify the relationship of urinary ANXA1 levels to renal pathological lesions. We classified the patients' specimens as group 1: primary GN: nonproliferative GN, including MCD, MGN, and FSGS; group 2: primary GN: proliferative GN, including IgAN and CrGN, and group 3: secondary GN, including DN and LN, as described previously [42, 43]. As shown in Figure 7, urine ANXA1 levels were significantly increased in these three groups compared to normal control (all *P* < 0.005). Moreover, urinary ANXA1 was significantly increased in group 3 (773.13 ± 144.96 U/mg Cr) compared to both group 1 (217.10 ± 40.62 U/mg Cr, *P* < 0.005) and group 2 (294.01 ± 71.89 U/mg Cr, *P* < 0.01). In all these three groups, urinary ANXA1 levels were significantly positively correlated with glomerular proliferation (group 1, *r* = 0.51, *P* < 0.01; group 2, *r* = 0.75, *P* < 0.005; group 3, *r* = 0.51, *P* < 0.05) and interstitial inflammation (group 1, *r* = 0.56, *P* < 0.005; group 2, *r* = 0.63, *P* < 0.05; group 3, *r* = 0.45, *P* < 0.05) (Table 2). In addition, the levels of urinary ANXA1 also correlated well with glomerular sclerosis in group 1 (*r* = 0.43, *P* < 0.01) and group 3 (*r* = 0.58, *P* < 0.05). Moreover, urinary ANXA1 levels were significantly positively correlated with crescentic formation in group 2 and group 3 (group 2, *r* = 0.69; group 3, *r* = 0.73) (both *P* < 0.05).

Combined all patients with various glomerular disorders, urinary ANXA1 protein levels positively correlated with the score of glomerular proliferation (*r* = 0.67, *P* < 0.001), interstitial inflammation (*r* = 0.54, *P* < 0.005), and glomerular sclerosis (*r* = 0.44, *P* < 0.001).

#### 3.6.3. As an Early Urinary Biomarker for Diabetic Nephropathy

Because diabetes is the most common single cause of end-stage renal disease (ESRD) in the world [44], we recruited 47 urine samples from diabetes patients without albuminuria (normoalbuminuria) or with early kidney involvement (microalbuminuria and macroalbuminuria) in this study. As shown in Table 3, the urinary ANXA1 was detectable as early as the stage of normoalbuminuria in diabetes patients with 52.94%, microalbuminuria with 72.73%, and macroalbuminuria with 100%.

## 4. Discussion

These findings support the value of the use of ANXA1 in various glomerular disorders as (1) a universal diagnostic and prognostic biomarker, (2) a noninvasive urinary early marker that can help in the differential diagnosis of the progressive types of glomerular disorders that can cause nephrotic syndrome from MCD, the most common, but favorable, type of glomerular disorder, and (3) a potential index for differentiating systemic diseases with kidney involvement from primary glomerular disorders.

Studies over the past 10 years have shown that the 37 kDa ANXA1 is cleaved at the N-terminus to generate the 33 kDa isoform, an event that occurs following the adhesion of blood neutrophils to the capillary endothelial cells, a process that activates protein externalization [45]. In the present study, both the 37 kDa intact ANXA1 and 33 kDa cleaved ANXA1 were detected in the urine (Figure 5). It has been demonstrated that the full-length ANXA1 and its cleaved form are released by apoptotic polymorphonuclear neutrophils and apoptotic mesangial cells [46]. Our data suggest that urinary ANXA1 comes from damaged and/or apoptotic renal tissues. ANXA1 became detectable in the urine as early as day 7 in the ADG model (Figure 5) and, importantly, was detectable in almost all urine samples from patients with glomerular disorders except those with MCD (less than 10.52% of cases were detected). Moreover, urinary levels of ANXA1 in secondary glomerular diseases, such as LN and DN, were significantly higher than those in primary glomerular disorders, including nonproliferative or proliferative GN (Figure 7), suggesting that ANXA1 may be helpful in the differential diagnosis of secondary versus primary glomerular disorders.

In fact, early detection of risk leading to the possibility of intervention before advanced renal injury has occurred is an obviously important goal. We use urine from diabetes patients, the most common single cause of ESRD in the world, to evaluate whether urinary ANXA1 could serve as an early biomarker for kidney injury. At present, microalbuminuria is considered the best available noninvasive marker for diabetes risk, but recently more and more studies have proved that it has inadequate specificity and sensitivity [47, 48]. In this study, we demonstrated that ANXA1 protein was earlier present in 52.94% of diabetes patients with normoalbuminuria. Thus, we infer that urinary ANXA1 may serve as an early diagnosis marker for glomerular injury.

ANXA1 has been reported to be highly expressed in epithelial cells of Bowman's capsule, macula densa, and medullary/papillary collecting ducts of the normal rat kidney [49] and overexpressed in multiple human cancers, including conventional renal cell carcinomas [33]. In the present study, we first demonstrated that podocytes, epithelial cells in the crescent, and parietal epithelial cells were the major sites of ANXA1 expression in the glomerulus, as well as certain actively regenerating renal tubules (Figures 3, 4, and 6).

Several reports have demonstrated that cytoplasmic ANXA1 can be induced to translocate into the nucleus by treatment with specific stimuli, such as EGF in the case of alveolar basal epithelial cells [50] and PMA in the case of human embryonic kidney cells [51], suggesting that ANXA1 nuclear translocation is involved in the regulation of cellular proliferation and may be directly involved in tumor invasion [52]. Although approximately 25% of ANXA1-positive cells in patients showed nuclear staining in addition to cytoplasmic staining, the significance of nuclear ANXA1 staining remains unclear and deserves further investigation.

We demonstrated here that ANXA1 may be involved in protecting glomerular proliferation and inflammation in glomerular injury. These findings from ISH and IHC data demonstrated that time-dependent enhancement in ANXA1 and the urinary levels of ANXA1 significantly correlate between the degree of glomerular proliferation and renal interstitial inflammation from histopathological assays, suggesting that ANXA1 plays an important inhibition role in renal injury processes. Both proliferation and inflammation are believed to play a major role in the progression of glomerular disorders [5, 53]. ANXA1 has been demonstrated to inhibit cell proliferation in several cell types, including mesangial cells [54], by inhibition of cyclin D1 expression through ERK1/2 MAPK signaling [55]. In contrast, overexpression of ANXA2 promotes cell proliferation in lung cancers [56] and inhibits apoptosis in breast cancers [57]. In addition, ANXA1 has been described as playing a homeostatic role in cells of the innate immune system [15, 58] and to act as a protective and anti-inflammatory protein in models of rheumatoid arthritis and myocardial infarct [15, 59, 60] by direct inhibition of phospholipase A2 [14]. Besides, ANXA2 can be detected in renal tissues from mice under normal status and renal injury [9, 10], and soluble ANXA2 tetramer has been reported to act as a soluble mediator of macrophage activation [19]. Fan et al. [61] demonstrated that ANXA1 and ANXA2 act as bridging molecules linking phagocyte and target cells and promote the phagocytosis of apoptotic lymphocytes, which results in reduction of inflammation. In the ADG model, we showed upregulation of ANXA1 and ANXA2 during the course of the disease, and this effect is consistent with our previous report of increased F4/80+ macrophage infiltration in the kidney in the mouse model [38]. Based on our observations and these published data, it is suggested that the very early enhanced renal expression of ANXA1 mRNA and protein in the ADG model might act to protect against glomerular proliferation and renal inflammation.

McArthur et al. [62] showed that ANXA1 exerts an autocrine-paracrine action leading to Rho kinase (ROCK) activation and actin polymerization. Rho/ROCK signaling has been linked to actin polymerization [63] and is involved in tubulointerstitial fibrosis [64] and glomerulosclerosis [65]. We also demonstrated that ANXA1 positively correlated with the degree of glomerular sclerosis in all patients with various types of glomerular disorders and crescentic formation in patients with primary GN: proliferative GN and secondary GN. Further studies on the roles of ANXA1 in renal fibrosis/sclerosis and in protecting renal tissues from proliferation and inflammation during the evolution of glomerular disorders are warranted. It is necessary to add other new techniques such as flow cytometry or multiplex assays in future investigation to evaluate the ANXA1 expression in the renal samples of patients for pursuing ANXA1 as a reliable biomarker with predictive and diagnostic values.

In summary, we demonstrated that (1) the detection of ANXA1 protein in the urine may be helpful as a noninvasive approach for the diagnosis and prognostic prediction of various glomerular injury; (2) ANXA1 can be used as a sensitive urinary biomarker in a rapid test allowing most forms of glomerular disorders to be differentiated from MCD; (3) ANXA1 shows potential for differentiating between secondary and primary glomerular disorders; and (4) ANXA1 and ANXA2 can serve as injury markers and may play roles in the persistence and progression of glomerular disorders.

## Supplementary Material

Characteristics of 47 diabetic patients without albuminuria (normoalbuminuria) or with early kidney involvement (microalbuminuria and macroalbuminuria).

## Figures and Tables

**Table 1:** Urine ANXA1 levels and urinary albumin excretion in patients with various types of glomerular disorder.

Diagnosis	U/mg Cr*	UACR* (mg/g)
MCD	0	172.68
IgAN	130.87	43.43
MGN	157.62	88.97
FSGS	382.18	105.53
CrGN	524.29	267.17
DN	563.27	215.65
LN	554.59	117.01
Normal control	0	2.5

*Data are median.

MCD: minimal change disease; MGN: membranous glomerulonephritis; FSGS: focal segmental glomerulosclerosis; IgAN: IgA nephropathy; CrGN: crescentic glomerulonephritis; DN: diabetic nephropathy; LN: lupus nephritis.

**Table 2:** Correlation of urinary ANXA1 levels with pathological findings in patients with various types of glomerular disorders.

	Primary GN					
	Nonproliferative GN		Proliferative GN		Secondary GN	
	*r *	*P *	*r *	*P *	*r *	*P *
Glomerular proliferation	0.51	0.008	0.75	0.004	0.51	0.043
Glomerular sclerosis	0.43	0.006	0.55	0.063	0.58	0.033
Interstitial inflammation	0.56	0.003	0.63	0.029	0.45	0.027
Interstitial fibrosis	−0.12	0.612	0.30	0.346	0.22	0.408
Crescentic formation	—	—	0.69	0.012	0.73	0.001

**Table 3:** Urine ANXA1 levels in diabetic patients without albuminuria or with early kidney involvement.

	U/mg Cr*	Percentage
Normoalbuminuria	3.17	52.94% (*n* = 14)
Microalbuminuria	4.90	72.73% (*n* = 11)
Macroalbuminuria	19.90	100% (*n* = 22)

*Data are median.
