# Supplementary material for: Urine Annexin A1 as an Index for Glomerular Injury in Patients
Source: Dis Markers. 2014 Jan 20;2014:854163. doi: 10.1155/2014/854163 (PMC3925619; doi:10.1155/2014/854163)
Supplement: Supplementary file 1 — Characteristics of 47 diabetic patients without albuminuria (normoalbuminuria) or with early kidney involvement (microalbuminuria and macroalbuminuria). [file 854163.f1.pdf]

Supplementary Table 1. Characteristics of 47 diabetic patients without albuminuria (normoalbuminuria) or with early kidney involvement (microalbuminuria and macroalbuminuria).

	**Normoalbuminuria**	**Microalbuminuria**	**Macroalbuminuria**
**Age (year)**	58 (43-72)	71 (51-81)	65.5 (49-75)
**BMI (kg/m^2)**	38.7 (32.5-49.1)	41.4 (37.5-51.9)	42.4 (37.2-46.3)
**SBP (mmHg)**	130 (120-150)	140 (118-160)	144 (120-170)
**DBP (mmHg)**	80 (70-92)	82 (70-92)	85 (70-100)
**HbA1c (%)**	7.1 (5.6-11.6)	7.5 (5.5-10.4)	8.45 (7.2-11.4)
**Total cholesterol (mmol/L)**	206.5 (144-300)	179 (142-284)	164.5 (149-213)
**Triglyceride (mmol/L)**	116 (61-508)	140.5 (71-494)	152.5 (82-270)
**GPT (U/L)**	31 (12-62)	20 (11-62)	20.5 (12-92)
**Serum creatinine (mg/dL)**	0.8 (0.6-1.2)	0.9 (0.6-1.3)	0.75 (0.7-1.1)
**UACR**	12.60 (3.4-19.97)	60.33 (24.01-291.67)	436.70 (312.82-1459.77)

Data are median (interquartile range).
